# A truth inference scheme for crowdsourcing using NLP and swin transformers

**DOI:** 10.1038/s41598-025-10942-x

**Published:** 2025-08-04

**Authors:** Ayswarya R. Kurup, Mithun Kumar Kar, Somila Hashunao, Madhusudhan Mishra

**Affiliations:** 1 Amrita School of Artificial Intelligence, Amrita Vishwa Vidyapeetham, Coimbatore, India; 2 Department of Electrical Engineering, North Eastern Regional Institute of Science and Technology (NERIST), Nirjuli, Arunachal Pradesh India; 3 Electronics and Communication Engineering, North Eastern Regional Institute of Science and Technology (NERIST), Nirjuli, Arunachal Pradesh India

**Keywords:** Crowdsourcing, Transfer learning, Truth inference, Natural language processing, Swin transformers, Computational science, Computer science, Information technology

## Abstract

Crowdsourcing has become a prevalent method for data collection across various domains, offering a scalable and cost-effective solution. However, ensuring the reliability of crowdsourced data remains a significant challenge due to the varying expertise of contributors and the complexity of tasks. Truth inference aims to derive high-quality and accurate answers from heterogeneous and noisy responses for crowdsourcing tasks. In order to address these challenges, we propose a truth inference model that integrates Natural Language Processing with transfer learning using Swin transformers. Unlike traditional transformer architectures, the Swin transformer employs a shifted windowing technique that effectively captures both local and global contextual features in textual data. This approach helps to generate more accurate embedding representations, specifically fine-tuned for nuances of crowdsourced tasks. By incorporating the Swin transformer, our model dynamically refines contributor reliability scores and task difficulty estimates, resulting in a more accurate truth inference. Experimental evaluations on multiple crowdsourcing datasets demonstrate that our approach consistently outperforms state-of-the-art methods in accuracy, scalability, and robustness, particularly under noisy and complex task conditions.

## Introduction

Crowdsourcing has emerged as a cost-effective strategy that uses collective intelligence to solve the problems challenging to automated processes^1^. Despite its vast applications, the reliability of crowdsourced data remains a challenge due to the heterogeneous nature of contributions, the ambiguity of tasks, and the presence of noisy labels. This makes the truth inference process difficult because accurate and reliable information should be derived from crowdsourced data. This is particularly significant when data integrity is crucial for decision-making and subsequent analytical processes^2^.

In crowdsourcing, truth inference aims to infer accurate and reliable responses for a set of tasks from contributor submissions. Traditional approaches for truth inference rely on methods such as majority voting^3–5^, Expectation-Maximization (EM) algorithms^6,7^, and Bayesian models^8,9^. However, these methods often fail to handle complex tasks, specifically when the expertise of contributors is unevenly distributed. For example, majority voting simply selects the response that the majority of contributors agree on. It assumes equal reliability for all contributors, which makes it ineffective when a large percentage of contributors lack domain expertise. In a medical annotation task, where 70% are inexperienced contributors and only 30% have domain expertise, majority voting is likely to yield incorrect results. Similarly, in sentiment analysis tasks, spammer contributors assign the same label for all inputs, so that the rewards are maximized with minimal effort. In such cases, majority voting amplifies the bias and reduces the overall data quality. Similarly, EM-based approaches assume that the contributor reliability is fixed without considering the task complexity. However, in dynamic crowdsourcing environments, contributor performance varies significantly over time based on the nature and difficulty of tasks. For example, a simple binary classification task and a complex language translation task which requires deep semantic understanding, assumed with uniform contributor reliability results in skewed and unreliable results. Furthermore, Bayesian models consider predefined prior distributions for computing the accuracy of responses. These priors may not always reflect real-world variations and result in less reliable truth inference. Moreover, these methods do not consider the context or credibility of contributors, which results in sub-optimal data quality.

Truth inference in crowdsourcing faces significant challenges due to the heterogeneous nature of crowdsourced contributors, whose diverse skill levels, domain expertise, and engagement patterns create substantial variance in response quality. These disparities, combined with varying task complexity, introduce systematic biases and inconsistent labeling patterns that compromise data reliability. Therefore, it is important to address these issues for improving the quality and accuracy of crowdsourced data, especially when tasks include both structured (e.g., data annotation) and unstructured (e.g., free-text responses) data. In this paper, we present a truth inference model that integrates Natural Language Processing (NLP) with the Swin transformers^10^ to improve the quality of submissions in crowdsourced environments. The use of NLP facilitates a deep, semantic understanding of textual data, helping the model to capture the contextual nuances from task descriptions. The Swin transformer introduces a shifted windowing mechanism, which allows the model to capture both local and global contextual information better compared to traditional transformers. By adapting the Swin transformer for crowdsourcing, the proposed model processes complex task descriptions and evaluates contributions with contextual understanding, resulting in more accurate truth inference.

The proposed truth inference model uses the hierarchical structure of the Swin transformer to refine the embeddings for truth inference. It processes the textual descriptions associated with crowdsourced tasks and generates contextual embeddings that capture key semantic features. These embeddings are refined iteratively with various optimization techniques to minimize the difference between predicted and true labels. This ensures that the model converges to an accurate representation of the underlying truth. This approach addresses the variability in data quality and the reliability of contributors, without the need for domain-specific training data. Next, these refined embeddings are combined with the contributor reliability scores and task difficulty levels to improve the inferred truth. The shifted windowing technique in the Swin transformer helps our model to capture relevant contextual information, even in large and complex datasets, thereby improving the accuracy and robustness of the inference process.

The truth inference process begins with preprocessing the text descriptions and labels from the crowdsourced responses. This ensures they are appropriately formatted for processing with the Swin transformer. The text is tokenized, and the embeddings are generated for each token to capture the semantic nuances of the descriptions. Unlike traditional models that use simple tokenization and embedding methods, the proposed approach benefits from the Swin transformer’s ability to generate rich, hierarchical embeddings through its shifted window mechanism. This mechanism segments the text into windows, which are then shifted and processed across multiple layers. This allows the model to capture both local and global context. Consequently, the model extracts more informative embeddings that are essential for accurate truth inference in crowdsourcing environments.

Subsequently, these embeddings are fed into a dynamic aggregation algorithm, which iteratively updates the estimated true labels by assessing the reliability of each contributor’s responses. This approach dynamically adjusts the weight factor of each contributor based on the quality and consistency of their inputs. It allows for more accurate and robust truth inference. Moreover, the algorithm updates the reliability scores of contributors based on their agreement with the current estimate of the true labels. This iterative process continues until the model converges on a stable set of true labels. This helps to mitigate the impact of unreliable or biased contributions, which improves the accuracy and reliability of the crowdsourced data.

The experiments with the proposed truth inference model demonstrate that it outperforms existing methods, providing more reliable results across both structured and unstructured tasks. Furthermore, our approach is designed to be both scalable and adaptable, making it suitable for a wide range of crowdsourcing tasks, regardless of the data type. The main contributions of this work are

i A novel truth inference model for crowdsourcing that combines NLP and transfer learning using Swin transformers to infer high-quality responses for both structured and unstructured tasks. This model is fine-tuned to address variability in data quality and contributor reliability.
ii The proposed model utilizes Swin transformer-based contextual embeddings and semantic analysis to achieve a deeper understanding of both structured and unstructured data. This integration improves the accuracy and reliability of crowdsourced data.
iii We validate the proposed framework through extensive experiments using multiple datasets collected from various crowdsourcing platforms. The results show substantial improvements in performance compared to existing truth inference methods.

The remainder of the paper is structured as follows. In “Literature review” section, we briefly review the relevant literature on crowdsourcing truth inference methods. In “The proposed truth inference model” section, we introduce the proposed truth inference model for crowdsourcing systems. The experimental details are discussed in “Experiments” section. Finally, we conclude in “Conclusion” section.

## Literature review

Truth inference is a significant process in crowdsourcing, to infer the correct responses from the diverse contributions from the crowd. Several truth inference models have been proposed, primarily focusing on methods such as majority voting, expectation maximization, and more sophisticated probabilistic models^2,11–13^.

Ipeirotis et al.^14^ have focused on algorithmic approaches to evaluate and infer the truthfulness of crowdsourced data based on redundancy and consensus techniques. However, these approaches fail when data is sparse or highly specialized, leading to inaccurate submissions.

Suyal et al.^15^ compared the existing truth inference algorithms for crowdsourcing and conducted a performance analysis on various datasets. They categorized the the truth inference methods into direct calculation methods, optimization methods, and probabilistic propagative methods. Also, they pointed out the necessity of developing algorithms that work on multi-class datasets and address the data sparsity in multi-class datasets.

Zheng et al.^16^ conducted an in-depth analysis of various truth inference methods. The analysis aims to understand how each of these methods handles different types of tasks and their difficulty level. They have developed a framework to categorize the methods based on their task types, various contributor models, and truth inference techniques. Further, they noticed that existing methods primarily focus on single-label, decision-making, and numeric tasks. More complex and unstructured task types, such as translation or sentiment analysis tasks, are less studied. It suggests a gap in how well current truth inference methods can be applied.

Probabilistic models helps to tackle the complexities of aggregating reliable information from the responses of crowd. Whitehill et al.^17^ proposed a model to estimate the true labels based on the reliability of the contributor and difficulty level of tasks. Similarly, Welinder et al.^18^ proposed a truth inference model that represents both annotator biases and competencies, and the ambiguity of images, in a multi-dimensional space. Further, Zhou et al.^19^ proposed the Minimax Conditional Entropy-based model that formulated the truth inference as an optimization problem. This model focuses on directly inferring the most probable true label by minimizing the expected loss, rather than explicitly estimating contributor reliability.

Transfer learning focuses on improving the learning in one task by transferring knowledge from a related task that is already learned. In crowdsourcing, this is particularly useful in addressing the scarcity of labeled data in specific tasks by leveraging knowledge from related crowdsourcing tasks. Several studies focused on domain adaptation, a subset of transfer learning, to mitigate issues arising from the variability in data quality and task specificity in crowdsourcing. Mo et al.^20^ proposed cross-task crowdsourcing that adapts models across various but related tasks, thereby improving the robustness of truth inference mechanisms.

Fang et al.^21^ and Han et al.^22^ illustrated the application of transfer learning to probabilistic models for truth inference. These models generate new instance representations from auxiliary information, thereby refining the accuracy of truth inference and assessing the expertise of workers. These representations help in better modeling of task complexity and predicting the reliability of the answers derived from crowdsourced data.

Zhang et al.^23^ proposed a meta-learning-based approach for truth inference in crowdsourced answers. They used a probabilistic approach that transfers meta-knowledge to high-level representations. These representations along with the instance features are used for inferring the correct labels. Zhong et al.^24^ proposed an approach based on the propagation of reliability in decisions and learning an ensemble of relevant graphs. The propagated reliability is then used for aggregating the answers from multiple sources. However, it is limited to categorical decisions, which restricts the model’s applicability to numerical decision-making tasks. It has complexity in the propagation of information reliability when applied to very large datasets or in real-time scenarios.

Li et al.^25^ proposed a label consistency-based approach that uses contributor reliability by evaluating the consistency of their labels across multiple tasks. They have improved the accuracy of truth inference by identifying the systematic labeling behavior and performing a label selection rather than label aggregation. This approach shows notable improvement over traditional probabilistic models by explicitly modeling the consistency of the contributors. However, it lacks in using deep semantic relationships and varying task complexities to improve the quality of the inferred labels. Li et al.^26^ developed a certainty-weighted voting-based algorithm that integrates contributor confidence levels into the truth inference and task aggregation process. This approach assigns weights to individual tasks based on the certainty of the contributors, thereby filtering out unreliable submissions.

Furthermore, Lin et al.^27^ used graph-based methods to capture the relationship between workers and tasks. This improves the accuracy of truth inference by considering the dependencies and interactions in crowdsourcing systems. Shao et al.^28^ proposed a Bayesian deep generative model that combines deep neural networks with a probabilistic structure for encoding uncertainties, achieving high accuracy in truth inference tasks. It leverages the strengths of deep learning for capturing complex patterns and the probabilistic nature of Bayesian inference to quantify uncertainty and improve the decision-making process. However, this approach requires extensive labeled data, which is challenging in many crowdsourcing scenarios. Also, the scalability of the large datasets is limited due to the computational overhead of a hybrid model.

Recent advances in transformer architectures have expanded the possibilities for natural language processing tasks, with potential applications to truth inference in crowdsourcing. While BERT-based models have been explored in some crowdsourcing scenarios^29^, other transformer variants offer unique capabilities that could benefit truth inference tasks. Dai et al.^30^ introduced Transformer-XL, with segment-level recurrence that could help maintain contextual coherence in lengthy crowdsourced documents. RoBERTa^31^ enhances BERT through dynamic masking, which could improve contributor reliability estimation. Zaheer et al.^32^ proposed Big Bird for efficiently handling longer sequences through sparse attention patterns which is valuable for processing extensive crowdsourced data. These architectures primarily focus on extending the context length or improving the general language understanding capabilities. Beyond natural language processing, transformer-based architectures have demonstrated effectiveness in adjacent reasoning tasks, such as robust visual question answering^33^. While these applications target different modalities, they highlight similar challenges in developing robust inference mechanisms for potentially noisy or ambiguous inputs.

Even though the existing works extensively explores the challenges of truth inference process in crowdsourcing, particularly through traditional machine learning and transformer-based models like BERT, there remains a gap in methods that effectively adapt to the dynamic and varied nature of crowdsourcing tasks. These models are limited by their dependence on extensive domain-specific training data and their difficulty in handling the intricate contextual subtleties inherent in complex datasets. Furthermore, these methods fail with the scalability or adaptability to new types of tasks, which is significant in diverse and rapidly evolving crowdsourcing environments. To address these limitations, it is essential to develop a more adaptable and robust approach to truth inference. Hence, we propose a model that combines NLP with transfer learning using the Swin transformers to achieve a deep semantic understanding of the structured and unstructured tasks. Through adapting the hierarchical and context-aware capabilities of the Swin transformers, our model dynamically adjusts to new data and feedback. This improves the accuracy and robustness of truth inference in crowdsourcing environments.

## The proposed truth inference model

A truth inference method aims to infer the accurate responses for a set of tasks, from the submissions provided by the contributors. The proposed truth inference model essentially captures the correct responses for structured and unstructured tasks. It uses NLP with transfer learning using the Swin transformer, while integrating the reliability assessment of contributors. The Swin transformer’s ability to derive hierarchical embeddings supports the semantic understanding of unstructured responses, while the task aggregation process enables consistent processing of structured responses. This approach consists of three main phases, including data preprocessing and feature extraction, transfer learning for domain adaptation, and the dynamic truth inference process.

**Table 1:** Table of notations.

Notation	Description
*T*	Set of tasks
*M*	Total number of tasks
$l_i$	True label for task $t_i$
*A*	Set of candidate answers
*L*	Set of true labels
*X*	Number of crowdsourced contributors
*N*	Set of noisy labels
*J*	Empirical loss
$D_i$	Textual description of task $t_i$
*W*	Sequence of words or tokens
$e_i$	Initial embedding for token $w_i$
*TE*	Token embedding
*SE*	Segment embedding
*PE*	Position embedding
$h_i^b$	Representation of token $w_i$ at layer *b*
*TB*	Transformer block
$c_i$	Contextualized representation of token $w_i$
*Q*	Queries in shifted window self-attention
*K*	Keys in shifted window self-attention
*V*	Values in shifted window self-attention
$\theta$	Parameters of Swin transformer model
$\eta$	Learning rate
$r_x$	Reliability score of contributor *x*

### Problem definition

Consider a crowdsourcing system comprising a set of *M* tasks, denoted as $T = \{ t_1, t_2, t_3,..t_M\}$, where each task $t_i$ contains an unknown true label $l_i$, which belongs to a set of candidate answers *A*. The complete set of true labels is $L = \{ l_i\}_{i=1}^M$. Additionally, *X* crowdsourced contributors provide noisy labels for the tasks. The noisy labels provided by contributor *x* for task $t_i$ are represented as $N = \{n_{x,i}\}$, where $x \in [1,X]$ and $i \in [1, M]$. Moreover, $\{n_{x,i}\}$ $\in$ *A*.

The primary objective is to develop a truth inference model capable of estimating the true labels *L*, for the set of tasks *T*, provided the noisy labeling data. The goal is to minimize the empirical loss

1 $$\begin{aligned} J = \frac{1}{M} \sum _{i = 1}^M {\mathbb {I}} ({\hat{l}}_i = l_i) \end{aligned}$$

where ${\mathbb {I}}$ is an indicator function that returns 1 if its argument is true and 0 otherwise, and $\hat{l_i}$ represents the model’s estimate of the true labels $l_i$.

**Fig. 1:**
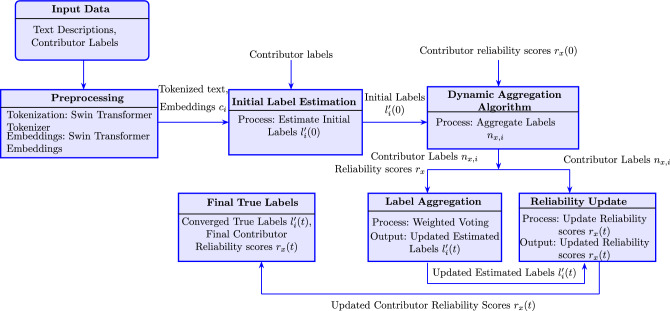
The proposed truth inference model using NLP and transfer learning with Swin transformer. The pipeline flows from top to bottom: (1) Input Data and Preprocessing generate embeddings from text; (2) Initial Label Estimation generates starting label estimates; (3) Dynamic Aggregation Algorithm iteratively refines labels through the Label Aggregation and Reliability Update processes; and (4) Final True Labels represent the converged outputs.

To address this, we integrate transfer learning and NLP, leveraging the advanced capabilities of the Swin transformer^10^. Transfer learning allows our model to dynamically adapt to new types of crowdsourcing tasks, facilitating efficient convergence and fine-tuning even with limited training data. This adaptability is important in crowdsourcing environments, where tasks are diverse and data can often be sparse or noisy. The Swin transformer, with its unique shifted windowing mechanism, improves the model’s ability to generate robust, contextually rich representations of the tasks. By combining NLP with transfer learning, our method significantly improves the effectiveness and accuracy of truth inference in various crowdsourcing tasks. Figure 1 illustrates the main processes in our proposed truth inference model. The notations used in our model are given in Table 1.

### The proposed framework using NLP and transfer learning with Swin transformer

This section outlines the proposed truth inference framework and its core components, including data preprocessing and feature extraction, transfer learning for domain adaptation, and the dynamic truth inference process.

#### Data preprocessing and feature extraction

Initially, for data preprocessing and feature extraction we use NLP techniques, specifically textual data embedding. Accurate truth inference from noisy crowdsourced data presents the critical challenge of effectively interpreting the textual descriptions provided for each task, whether structured or unstructured. To address this, we use the Swin transformer, which is renowned for its ability to capture both local and global contextual information through its unique shifted windowing mechanism.

The Swin transformer encodes text into a high-dimensional space, capturing the hierarchical and semantic features that are significant for understanding the subtleties of the data, which is essential for accurate label prediction. These embeddings are iteratively refined using optimization techniques to minimize the discrepancy between predicted and true labels. This ensures that the model converges to accurately represent the underlying truth. Each task $t_i$ is associated with a textual description $D_i$, which is the input to the Swin transformer. This text is tokenized into a sequence of words or tokens $W = [w_1, w_2,\ldots , w_n ]$. Next, the Swin transformer processes these tokens, and generates initial embeddings $e_i$. These embeddings encapsulates the semantic features which are necessary for understanding the task through its multi-layered processing approach.

2 $$\begin{aligned} e_i = TE(w_i) + SE(w_i) + PE(i) \end{aligned}$$

where $TE(w_i)$ represents the token embedding, which captures the intrinsic meaning of the word. $SE(w_i)$ is the segment embedding that distinguishes different sentences or segments in the input. *PE*(*i*) represents the position embedding that encodes the position of the token in the sequence, which allows our model to retain the order of tokens.

Given the diverse and noisy nature of crowdsourced tasks, the Swin transformer’s ability to generate rich, hierarchical embeddings is significant for processing structured and unstructured data. After initializing the token embeddings, they are passed through the Swin transformer. The Swin transformer uses a shifted windowing technique, which divides the sequence into smaller windows and processes them independently. In subsequent layers, these windows are shifted to overlap, which allows the model to integrate information across different parts of the sequence. This hierarchical processing is particularly advantageous for crowdsourcing tasks, where a detailed understanding (local context) and an overarching view (global context) of the textual descriptions are significant for accurate truth inference. Finally, the Swin transformer generates contextually rich embeddings that manage the complexity of unstructured data, while ensuring consistency in processing structured data.

##### Shifted window multihead self-attention mechanism (SW-MSA)

While using the shifted window technique, the Swin transformer applies a self-attention mechanism within each window. This allows the model to focus on the most relevant tokens when constructing the representation of each token $w_i$. Consequently, the self-attention is computed as

3 $$\begin{aligned} \text {SW-MSA} ({\textbf {Q, K, V}}) = \text {softmax}\left( \dfrac{{\textbf {QK}}^T}{\sqrt{d_k}}\right) {\textbf {V}} \end{aligned}$$

where *Q*, *K*, and *V* represent the queries, keys, and values derived from the input embeddings, and $d_k$ is the dimensionality of the keys. The self-attention operation computes the relevance of each token to every other token within the window, facilitating the model’s ability to capture intricate dependencies and relationships within the text, which is essential for interpreting complex and diverse crowdsourced data.

##### Feed-forward network with Swish activation

Following the self-attention mechanism, the Swin transformer applies a feed-forward neural network (FFN) to each token’s representation. Furthermore, the FFN refines these representations by introducing non-linearity and enabling the model to learn complex functions that improve the semantic depth of the embeddings. To achieve this, within this FFN, we use the Swish activation function^34^ that is derived using

4 $$\begin{aligned} A_{Swish}(x) = x \cdot \sigma (x) = \frac{x}{1 + e^{-x}} \end{aligned}$$

where $\sigma (x)$ is the sigmoid function. We use the Swish activation function in our model due to its smooth, non-linear properties. This improves the gradient flow during backpropagation. This results in more effective weight updates and faster convergence during training. Moreover, this strengthens the model’s ability to capture intricate patterns in the data, thus making it particularly effective for complex data in crowdsourcing tasks.

After the non-linear refinement, the Swin transformer processes these token representations through a series of layers. The operation of a single Swin transformer block $TB_{Swin}$, on an output embedding from the previous layer ${\textbf {h}}_i^{b-1}$ is formalized as

5 $$\begin{aligned} {\textbf {h}}_i^b = TB_{Swin}({\textbf {h}}_i^{b-1}) \end{aligned}$$

where ${\textbf {h}}_i^0 = e_i$ is the initial embeddings, and *b* refers to the specific layer within the transformer.

The final output from the Swin transformer, $c_i = {\textbf {h}}i^B$, provides a rich, contextualized representation of each token embedding. This approach helps our model to address the variability in the quality of data and reliability of the contributors without extensive domain-specific training data.

Moreover, for tasks that require a comprehensive understanding of the textual descriptions, the embedding of a special classification token is used, which is denoted as CLS, and it is placed at the beginning of each sequence. This embedding, $c_i = {\textbf {h}} {CLS}^B$ encapsulates the aggregated information from the entire sequence. It is subsequently used in our dynamic truth inference model. This contextualized embedding is integrated with a dynamic weighting method that evaluates and incorporates the reliability of each contributor to accurately infer the true labels $l_i$. This method with the Swin transformer incorporated by the Swish activation function, essentially improves the robustness of the proposed truth inference approach, even in noisy and heterogeneous crowdsourced data.

#### Transfer learning for domain adaptation

In our approach, the pre-trained Swin transformer model is fine-tuned on a subset of the crowdsourced data that has been partially validated. To achieve this, transfer learning is incorporated into the proposed truth inference model through a two-step process, initializing with a pre-trained Swin transformer model. This helps to learn generalized features and representations. Next, we fine-tune this model on a task-specific dataset. This methodology helps to use the hierarchical and contextual understanding capabilities of the Swin transformer, which is originally trained on large-scale general data, and adapt it to the specific features of crowdsourcing tasks.

The fine-tuning process allows the Swin transformer to process both structured aspects of the dataset, such as predefined labels or discrete responses, and unstructured data, which includes complex text descriptions. This enables our model to effectively bridge the gap between general language understanding and the specific needs of crowdsourced truth inference.

For example, consider a task from the WikiSQL dataset, which consists of a set of questions and their corresponding SQL queries. During the fine-tuning process of the Swin transformer on this dataset, structured SQL queries represent the target responses, while the natural language questions serve as the unstructured input data. Hence, the model adapts by understanding the syntactic structure of SQL as well as the semantic context provided by the questions. This helps the Swin transformer to better understand database-related tasks and domain-specific language patterns relevant to SQL generation.

After fine-tuning, for a sequence of tokens $W = w_1, w_2,..w_n$ associated with a task $t_i$, the Swin transformer $T_{Swin}$ processes the sequence to generate contextualized embeddings as follows

6 $$\begin{aligned} {\textbf {h}}^p = T_{Swin}(S, \theta ) \end{aligned}$$

These embeddings are essential for inferring the true answers from the crowdsourced tasks. For instance, in the WikiSQL task, it captures both the syntactic and semantic nuances required for SQL generation, ensuring that it accurately infers the correct SQL query based on the contributor-provided labels.

In general, the dataset comprises pairs of textual descriptions and their associated true labels, $(W_i, l_i )$. The fine-tuning process involves adjusting the parameters $\theta$ of the Swin transformer on the task-specific dataset on $D_t$ to minimize the loss between the predicted labels and the true labels. This optimization can be represented as

7 $$\begin{aligned} \theta ' = \arg \min _\theta \sum _{(W_i, l_i) \in D_t} {\mathcal {L}}(T_{Swin}(W_i, \theta ), l_i) \end{aligned}$$

where *L* measures the discrepancy between the predicted labels and the actual labels, encouraging the model to adjust $\theta$ to $\theta '$ thereby better fitting the specific characteristics of crowdsourcing tasks. The parameters $\theta$ are updated iteratively through gradient descent optimization. In the $i^{th}$ round of optimization for each batch, $\theta$ is updated using the following gradient descent step

8 $$\begin{aligned} \theta \leftarrow \theta - \alpha \nabla _\theta \sum _{T_i} {\mathcal {L}}_{t_i}(f_\theta ) \end{aligned}$$

where the function $f_\theta$ represents the Swin transformer model parameterized by $\theta$. The learning rate $\alpha$ is the hyper parameter, controlling the step size in each iteration. This process minimizes the loss function ${\mathcal {L}}$ by iteratively updating $\theta$ until the convergence.

After fine-tuning, the Swin transformer’s output embeddings $c_i$ are passed as the features in our truth inference model. These contextually enriched embeddings, enhanced by the Swish activation function, are integrated with the reliability scores of crowdsourced contributors to infer the most probable true label $l_i$. This integration ensures that the model not only captures the semantic richness of the task descriptions but also effectively incorporates the varying reliability of each contribution, thereby improving the overall accuracy of the truth inference process.

**Fig. 2:**
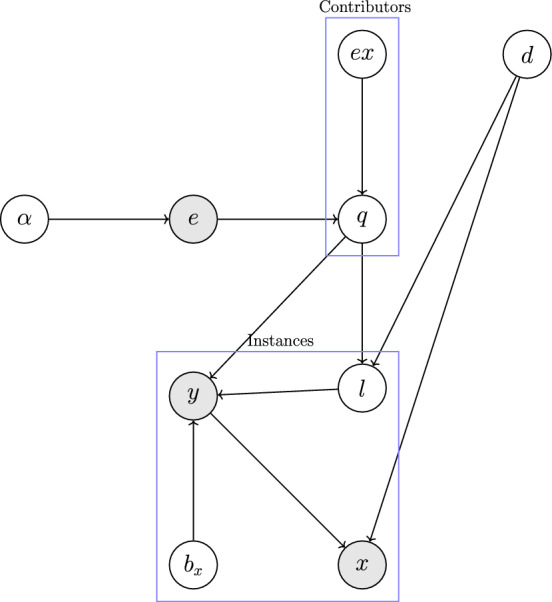
The probabilistic graphical model of the proposed Dynamic Truth Inference model. The shaded nodes represent the observed variables (crowdsourced label *y*, task instance *x*, and Swin transformer embedding *e*). The true label *l* of an instance, the contributor expertise *ex*, and the task difficulty *d* of an instance are hidden variables.

**Fig. 3:**
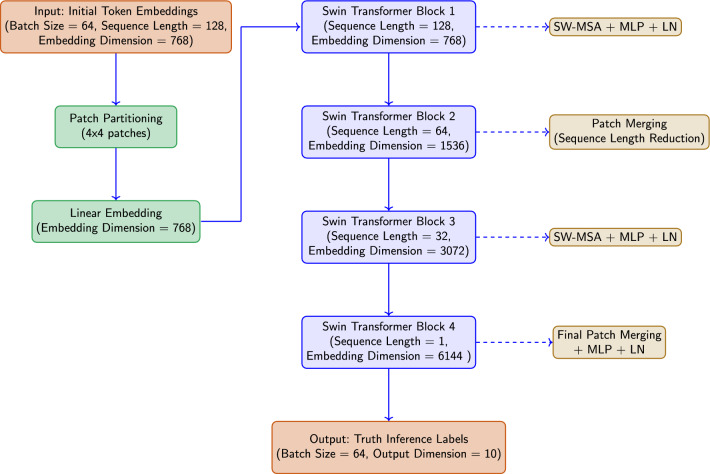
The schematic representation of the proposed truth inference model using Swin transformers. The architecture processes input token embeddings through a hierarchy of Swin Transformer blocks, which reduce sequence length while increasing feature dimensionality. Each block contains SW-MSA and MLP components with layer normalization (LN). Patch merging operations between blocks reduce spatial dimensions while doubling feature channels. The final patch merging and MLP layers produce the output truth inference labels.

#### The truth inference process

The dynamic aggregation algorithm evaluates the reliability of contributor-provided labels using the contextual embeddings generated by the fine-tuned Swin transformer model. We use the reliability score of each contributor $r_i$ is utilized in conjunction with the embeddings $c_i$ to estimate the correct labels for the tasks.

For each task $t_i$ with a textual description $D_i$, the fine-tuned Swin transformer generates contextual embeddings $c_i$. These embeddings capture both local and global contextual information, providing a rich representation of the task’s textual description. Contributors provide labels $l_{x,i}$ for each task, where *x* indexes the contributors. The dynamic aggregation algorithm integrates these labels with the reliability scores of contributors to iteratively refine the estimation of the true labels $l_i$.

Our algorithm uses the contextual embeddings generated from both structured and unstructured inputs to refine the contributor reliability scores and the inferred true labels. This combination enables a comprehensive evaluation, considering the consistency of structured labels alongside the semantic depth captured from unstructured descriptions. By incorporating both data types, the model can handle complex, diverse tasks which is essential for crowdsourcing tasks.

The core idea of this process is to dynamically adjust the influence of each contributor’s responses based on their reliability score $r_x$. We use the contextual richness of the Swin transformer-generated embeddings $c_i$, along with the Swish activation function to accurately assess the consistency and reliability of the contributor labels $l_{x,i}$. This approach ensures that more reliable contributors have a greater impact on the final inferred labels, thereby improving the overall accuracy of the truth inference process.

We begin with an initial estimation of the true label $l'_i(0)$ for each task, which is derived from the labels provided by all contributors. Each contributor $x$ is assigned a reliability score $r_x(t)$ based on their agreement with the initial true label estimation as

9 $$\begin{aligned} r_x(t) = \frac{1}{M} \sum _{i=1}^{M} \delta (n_{x,i}, l'_i(0)) \end{aligned}$$

where $\delta$ is the Kronecker delta function, which equals $1$ if the contributor’s label matches the initial estimated true label, and $0$ otherwise. Subsequently, the estimated true labels $l'_i(t)$ are updated iteratively using a weighted voting mechanism. For each task, the true label $l'_i(t)$ is updated by aggregating inputs from all contributors, weighted by their current reliability scores $r_x(t-1)$ using

10 $$\begin{aligned} l'_i(t) = \arg \max _{l \in L} \sum _{x=1}^{X} r_x(t-1) \cdot \delta (n_{x,i}, l) \end{aligned}$$

Here, *I* ranges over all possible labels in $L$, and $t$ denotes the iteration number. After each iteration, we update the reliability scores of contributors based on the latest estimate of the true labels $l'_i(t)$ as

11 $$\begin{aligned} r_x(t) = \frac{1}{M} \sum _{i=1}^{M} \delta (n_{x,i}, l'_i(t)) \end{aligned}$$

Next, the convergence of this process is updated by checking if the changes in $l'_i$ between iterations are below a specified threshold. It is given as

12 $$\begin{aligned} \sum _{i=1}^{M} \delta (l'_i(t), l'_i(t-1)) < \epsilon \end{aligned}$$

where $\epsilon$ is a small positive number. Once the convergence criterion is met, the final set of estimated true labels $L' = \{l'_i\}$ is considered to represent the actual truth, based on the aggregated information and the reliability of the contributors. The proposed truth inference process is detailed in Algorithm 1.

**Algorithm 1:**
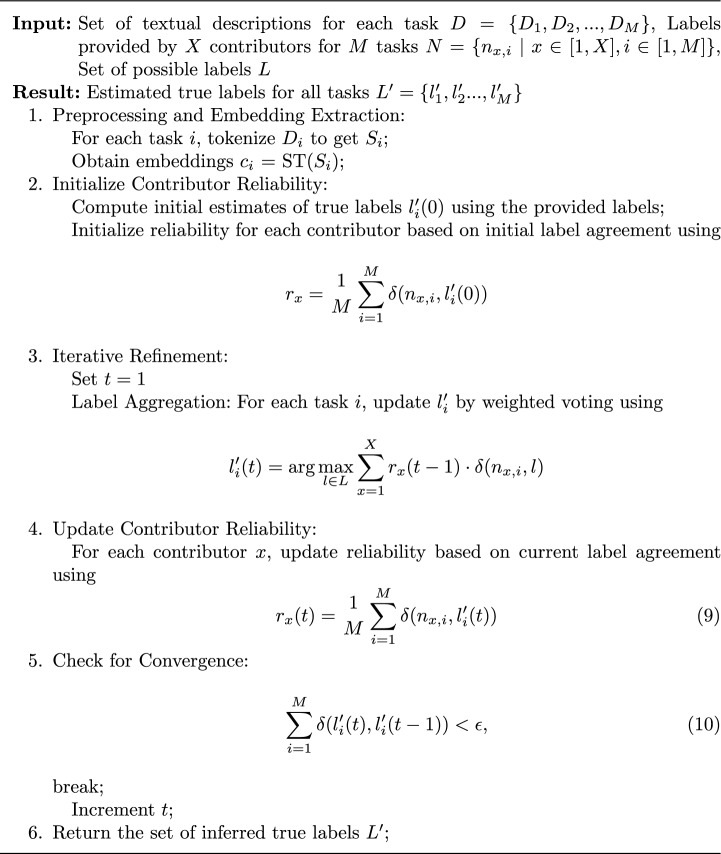
The proposed truth inference process.

Figure 2 illustrates the graphical model of our approach, where the observed variables include crowdsourced label *y*, task instance *x*, and Swin transformer embedding *e*. These embeddings *e* serve as input to the model, providing contextual information about the task descriptions. The latent variables such as the true label *l*, contributor expertise *ex*, task difficulty *d*, and the contributor-specific bias $b_x$ are inferred through the model’s probabilistic structure.

The true label *l* of an instance is influenced by both the intermediate representation *q*, derived from the embeddings, and the difficulty of the task *d*. Contributor expertise *ex* helps the model to learn the reliability of the contributor-provided labels, and $b_x$ is the local bias that captures the variability in individual contributor tendencies in the labeling process.

Figure 3 illustrates the schematic representation of the proposed truth inference model using Swin transformers. The process begins with the initial token embeddings which are derived from the raw text data. Each input sequence is represented as

$$\begin{aligned} E = [e_1,e_2, \ldots ,e_N] + PE \end{aligned}$$

where $E_0 \in {\mathbb {R}}^{L \times D}$ represents the initial token embeddings for sequence length *L* and embedding dimension *D*. Here, $e_i \in {\mathbb {R}}^D$ is the embedding of the $i^{th}$ token, and $PE \in {\mathbb {R}}^{L \times D}$ is the positional encoding, which preserves the order of tokens in the sequence.

During the patch partitioning stage, the sequence embeddings $E_0$ are segmented into smaller non-overlapping patches. This process helps to reshape the input into a set of patches, which is essential for capturing the local context within each patch. The partitioned patches $P_0$ are represented as

13 $$\begin{aligned} P_0 = P_{Swin} (E_0) \end{aligned}$$

where $P_0 \in {\mathbb {R}}^{L' \times D'}$ is the partitioned output after applying the Swin transformer partitioning function $P_{Swin}$, $L^{\prime}$ is the reduced sequence length, and $D^{\prime}$ is the embedding dimension.

Next, the linear embedding layer transforms each patch into a higher-dimensional space, maintaining the same dimension across the entire sequence. This linear transformation is essential for aligning the input dimensions with the subsequent Swin transformer blocks. Then, the output of the linear embedding is represented as

14 $$\begin{aligned} P_1 = \text {LE} (P_0) \end{aligned}$$

where $P_1 \in {\mathbb {R}}^{L' \times D'}$ is the output after applying the linear embedding.

These embeddings serve as the foundational input to our model, capturing both local and global contextual information from the raw text. As the data progresses through various stages of the Swin transformer blocks, the model refines these embeddings by incorporating semantic relationships and hierarchical structures. In the first Swin transformer block, the partitioned patches $P_1$ undergo the Shifted Window Multi-Head Self-Attention (SW-MSA) mechanism. This mechanism applies self-attention within each window of patches, where the window is shifted to capture cross-window interactions as given in Eq. (3), where the values *V* are derived from the input patches $P_1$. This operation results in an intermediate representation $P_2$, which retains the sequence length $L'$ and embedding dimension $D'$.

Next, the Swish activation function is applied within the MLP to introduce non-linearity which is given by

$$\begin{aligned} P_2 = \text {SW-MSA}(P_1)+ A_{Swish}(\text {MLP}(LN_{Swin}(P_1))) \end{aligned}$$

where the activation function is defined using Eq. (4), and $LN_{Swin}$ represents the Layer Normalization. This improves the model’s ability to capture complex patterns and improves gradient flow during training.

In the second Swin transformer block, the sequence undergoes patch merging, which reduces the sequence length while doubling the embedding dimension. This is necessary for capturing higher-level abstractions in the data. The new representation $P_3$ is given by

$$\begin{aligned} P_3 = M_{Swin}(P_2) \end{aligned}$$

where $P_3 \in {\mathbb {R}}^{L'' \times D''}$ and $M_{Swin}$ represents the patch merging operation. Further, the SW-MSA mechanism is applied again which is followed by MLP, Layer Normalization $LN_{Swin}$, and the Swish activation function $A_{Swin}$.

Subsequently, in the third and fourth Swin transformer blocks, the model continues to refine the sequence representation through an iterative process of patch merging, Shifted Window Multi-Head Self-Attention (SW-MSA), and non-linear transformations. These blocks reduce the sequence length while increasing the embedding dimension to capture higher-level abstractions and global context. In general, the patch merging is defined as

15 $$\begin{aligned} P_{k+1} = M_{Swin}(P_k) \end{aligned}$$

where *k* denotes the current block and $P_{(k+1)} \in {\mathbb {R}}^{L_{k+1} \times D_{k+1}}$ represents the merged patches. The merged patches are processed using the SW-MSA mechanism to capture both local and cross-window interactions which are represented as

16 $$\begin{aligned} P_{k+2}=\text {SW-MSA}(P_{k+1})+ A_{Swish}(\text {MLP}(LN_{Swin}(P_{k+1}))) \end{aligned}$$

where, MLP is a multi-layer perceptron, and $A_{Swish}$ is the activation function used to introduce non-linearity. After the sequence has been processed through the Swin transformer blocks, the final output $P_n$ from the last Swin transformer block represents a highly refined and contextually enriched embedding. This embedding captures the cumulative information from both local and global dependencies across the entire input sequence. Next, the $P_n$ is passed through a fully connected classifier layer which applies the Swish activation function to ensure smooth and efficient gradient flow, to capture complex patterns in the data. Then, the classifier translates the dense, context-rich embedding $P_n$ into a set of probabilities that correspond to the potential truth labels, which are then further processed in the dynamic truth inference stage.

Algorithm 2 describes the training algorithm used in our truth inference model to optimize the model to accurately infer truth from noisy and diverse crowdsourced data. This algorithm refines the model parameters using a combination of Swin transformers for contextual embeddings and dynamic weighting to adjust for contributor reliability. The process begins by initializing the model parameters, including the Swin transformer weights pre-trained on a large-scale dataset. Then, we apply initial randomization to the weights specific to the truth inference tasks. Next, the input textual data is tokenized, and the initial embeddings are generated using the Swin transformer. These embeddings capture both local and global context through the shifted windowing technique.

Further, for each batch of tasks, we compute the loss function ${\mathcal {L}}$ using the predicted and true labels. Then, the backpropagation is used to compute gradients and update the model parameters using gradient descent. Next, contributor reliability scores are dynamically updated by adjusting the weight of each contributor’s input based on their performance relative to the current model predictions.

Moreover, we periodically validate the model on a validation set to adjust hyper-parameters, such as the learning rate, based on validation performance to ensure optimal training progression. Then, a convergence check is performed to monitor the model’s performance metrics during training iterations. The training process continues until the change in these metrics falls below a predefined threshold, indicating convergence. Finally, upon convergence, select the final model based on the best performance on the validation set, ensuring that it has not only high accuracy but also generalizes well to unseen data.

**Algorithm 2:**
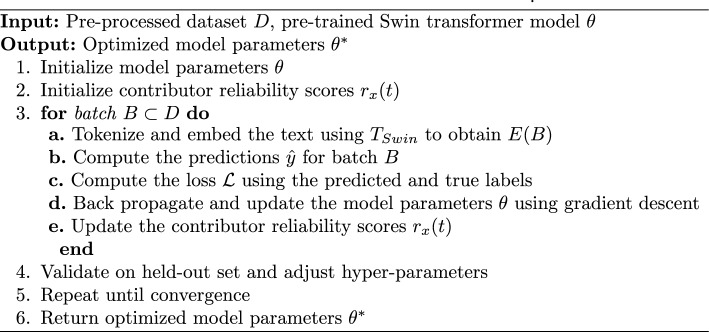
Training algorithm for the truth inference model.

## Experiments

In this section, we validate the effectiveness of our proposed dynamic truth inference model through a series of experiments. We detail the experimental settings, dataset, evaluation metrics, and baseline algorithms used for the validation^35^.

### Dataset

To comprehensively evaluate our model, we have selected three diverse datasets collected from Figure Eight^36^, WikiSQL^37,38^, and Amazon Mechanical Turk (MTurk)^39,40^. Additionally, we have generated synthetic data to further test the model’s scalability and robustness.

The Figure Eight dataset primarily contains tasks focused on classification. Each task is associated with a textual description averaging 50 words in length. A pool of 500 contributors provides both binary and multi-class labels for these tasks. The diversity in labeling makes this dataset particularly suitable for testing the model’s performance in handling varied label distributions, structured responses, and noisy inputs.

The WikiSQL dataset involves SQL query generation tasks, where contributors generate or validate SQL queries based on textual descriptions. Each task has an average description length of 70 words, and labeling contributions from 300 contributors. This dataset is challenging due to the complexity of SQL queries and the need for precise label validation, thus making it ideal for benchmark for evaluating how the proposed model adapts to structured and unstructured data.

The Amazon MTurk dataset comprises sentiment analysis tasks. It includes 15,000 tasks with responses from 400 contributors. The variability in contributor annotations makes this dataset ideal for evaluating the model’s ability to handle noisy and subjective labels.

Additionally, the synthetic data consists of tasks from question answering (QA), classification, and sentiment analysis. This dataset includes 25,000 tasks with responses from 600 contributors. The synthetic data is designed to test the scalability of the model and its performance in a controlled environment where task difficulty and contributor reliability can be systematically varied.

The dataset is preprocessed to align with our truth inference model’s input requirements. Initially, we have tokenized each task’s textual description using the Swin transformer’s tokenizer. This step involves segmenting the text into a sequence of tokens, which are then processed to capture both local and global contextual information. Next, the initial embeddings are generated for each token using the Swin transformer’s embedding layers. These embeddings are designed to capture the hierarchical semantic features of the text, providing a rich representation that is essential for accurate truth inference. Subsequently, these embeddings are fed into the dynamic aggregation algorithm to infer the true labels. The statistics of the dataset are provided in Table 2.

**Table 2:** Dataset characteristics.

Dataset	Number of tasks	Task types	Average task length	Number of contributors	Label types
Figure Eight	10,000	Classification	50 words	500	Binary, Multi-class
WikiSQL	20,000	SQL query generation	70 words	300	Structured, Text
MTurk	15,000	Sentiment analysis	40 words	400	Binary, Multi-class
Synthetic data	25,000	Various (QA, Classification, Sentiment Analysis)	60 words	600	Binary, Multi-class

### Hyper-parameters and training

We have fine-tuned the Swin transformer model with optimized hyper-parameters to improve the performance on crowdsourcing tasks. The proposed model is trained for 50 epochs. This duration is chosen based on the preliminary experiments and validation performance, to prevent overfitting. For regularization, the AdamW optimizer^41^ is used, which is well-suited for handling sparse gradients and incorporates weight decay correction. The maximum sequence length is set to 128 tokens. This allows the model to handle moderately long text inputs, typical of our datasets while maintaining computational efficiency.

The choice of hyper-parameters is determined through grid search and cross-validation^42^. Grid search systematically explores a range of hyper-parameter values, where the optimal configuration selected as, learning rate $3e-5$, batch size 16, dropout rate 0.1, and maximum sequence length 128 tokens. These values were validated through cross-validation to ensure that the selected parameters generalize across different data splits. This tuning process is essential for maximizing the model’s performance and ensuring robust, reliable results across our truth inference tasks. Moreover, to ensure the reliability of the experimental results, we have performed 5 independent runs for each experiment with different random seeds (42, 123, 456, 789, 999). The statistical significance is assessed using paired t-tests between the proposed TIA model and the baseline methods, with a significance threshold of $p < 0.05$.

### Evaluation metrics

We use the evaluation metrics accuracy, F1-Score, and AUC-ROC for evaluating the effectiveness of the proposed truth inference model. Accuracy defines the proportion of tasks for which the inferred labels match the true labels, and it is computed as

$$\begin{aligned} \text {Accuracy} = \dfrac{\text {Number of correct predictions}}{\text {Total number of predictions}} \end{aligned}$$

In addition to this, we use F1-Score, which is the mean of precision and recall.

$$\begin{aligned} \text {F1-Score} = 2 \times \frac{\text {Precision} \times \text {Recall}}{\text {Precision} + \text {Recall}} \end{aligned}$$

Another metric we have considered is AUC-ROC which measures the area under the receiver operating characteristic curve. It is computed by the true positive rate (TPR) and the false positive rate (FPR) at various threshold settings.

### Baselines

We compare our truth inference model with the following baselines.

- Majority Voting (MV)^3^: A conventional method that selects the label with the highest number of votes.
- DLC^43^: A CNN-based approach that processes contributor information using a crowd process layer.
- ALKT^21^: An active learning-based approach that uses a probabilistic graphical model to model the expertise level of contributors.
- Dawid-Skene (DS)^11^: A probabilistic model for aggregating crowdsourced labels.
- BERT without Transfer Learning (BWT)^29^: A BERT-based model for truth inference without domain-specific fine-tuning.
- LCTI^25^: A probabilistic graphical model that estimates the contributor reliability based on label consistency across multiple tasks.
- CWV^26^: A machine learning approach that applies certainty-weighted voting.
- CRMT^23^: A probabilistic model which integrates few-shot-meta learning and transfer learning techniques.
- Swin transformer without Transfer Learning (SWT): Our Swin transformer-based model without domain-specific fine-tuning.

### Results

In this section, we discuss the results of the experiments conducted on various datasets. The proposed truth inference model is abbreviated as TIA. Table 3 shows the performance of our model in terms of accuracy, precision, recall, F1-Score, and AUC-ROC on the Figure Eight dataset. For each baseline, the accuracy is computed by averaging the results of 5 runs. The results demonstrate that TIA consistently achieves better performance in predicting correct labels, achieving an accuracy of 87% and an F1-Score of 0.88. This substantial improvement over the baseline methods underscores the significance of incorporating transfer learning and deep semantic understanding through NLP-based contextual representations and Swin transformers, along with transfer learning capabilities. These components enable TIA to generalize across various tasks and model varying contributor behavior more effectively.

**Table 3:** Comparison results of the proposed model TIA and the baseline methods on the Figure Eight dataset.

Method	Accuracy	Precision	Recall	F1-Score	AUC-ROC	*p*-value
MV	0.71 ± 0.018	0.70 ± 0.021	0.75 ± 0.019	0.72 ± 0.017	0.70 ± 0.022	<0.001
DLC	0.745 ± 0.016	0.73 ± 0.018	0.81 ± 0.015	0.763 ± 0.014	0.78 ± 0.017	<0.001
ALKT	0.814 ± 0.012	0.77 ± 0.015	0.82 ± 0.013	0.794 ± 0.011	0.77 ± 0.016	0.002
DS	0.78 ± 0.019	0.76 ± 0.020	0.80 ± 0.018	0.78 ± 0.017	0.76 ± 0.021	<0.001
BWT	0.82 ± 0.011	0.80 ± 0.013	0.84 ± 0.012	0.82 ± 0.010	0.80 ± 0.014	0.003
LCTI	0.79 ± 0.015	0.77 ± 0.017	0.81 ± 0.016	0.79 ± 0.014	0.78 ± 0.018	<0.001
CWV	0.81 ± 0.013	0.79 ± 0.016	0.83 ± 0.014	0.81 ± 0.015	0.79 ± 0.015	0.001
CRMT	0.85 ± 0.009	0.83 ± 0.011	0.87 ± 0.010	0.85 ± 0.008	0.84 ± 0.012	0.015
SWT	0.82 ± 0.012	0.80 ± 0.014	0.84 ± 0.013	0.82 ± 0.011	0.80 ± 0.015	0.003
TIA	**0.87 ± 0.008**	**0.86 ± 0.010**	**0.90 ± 0.009**	**0.88 ± 0.007**	**0.87 ± 0.010**	–

The values in bold are observations from the proposed model.

Among the baselines, deep learning-based models such as BWT and SWT, as well as the meta-learning-based approach CRMT, show more competitive results than traditional probabilistic methods. This emphasizes the advantage of capturing deep contextual features and inter-task learning. However, these methods lack the cross-task knowledge transfer and contextual understanding, which is offered by TIA. Furthermore, CRMT performs competitively, which indicates that meta-knowledge contributes positively to truth inference. However, it is not as effective as the domain-specific fine-tuning and language modeling capabilities embedded in TIA.

Likewise, LCTI shows notable performance over traditional methods like DS by modeling annotator reliability through label consistency, but it lags behind deep learning methods in processing complex task features. In addition, CWV provides moderate improvement over models like MV, which uses simple voting schemes, by incorporating the contributor’s confidence levels into the task aggregation process. However, its performance is limited, since it does not capture the latent structure of contributor behavior or task complexity.

**Table 4:** Results of TIA and baseline methods on the WikiSQL dataset.

Method	Accuracy	Precision	Recall	F1-score	AUC-ROC	*p*-value
MV	0.70 ± 0.020	0.68 ± 0.022	0.72 ± 0.021	0.70 ± 0.019	0.68 ± 0.023	<0.001
DLC	0.79 ± 0.017	0.75 ± 0.019	0.79 ± 0.018	0.77 ± 0.016	0.75 ± 0.020	<0.001
ALKT	0.80 ± 0.014	0.74 ± 0.016	0.78 ± 0.015	0.76 ± 0.013	0.74 ± 0.017	<0.001
DS	0.75 ± 0.018	0.73 ± 0.020	0.77 ± 0.019	0.75 ± 0.017	0.73 ± 0.021	<0.001
BWT	0.81 ± 0.012	0.79 ± 0.014	0.83 ± 0.013	0.81 ± 0.011	0.80 ± 0.015	0.002
LCTI	0.78 ± 0.016	0.76 ± 0.018	0.79 ± 0.017	0.77 ± 0.015	0.75 ± 0.019	<0.001
CWV	0.77 ± 0.015	0.74 ± 0.017	0.78 ± 0.016	0.76 ± 0.014	0.74 ± 0.018	<0.001
CRMT	0.82 ± 0.010	0.80 ± 0.012	0.83 ± 0.011	0.81 ± 0.009	0.79 ± 0.013	0.003
SWT	0.81 ± 0.013	0.79 ± 0.015	0.83 ± 0.014	0.81 ± 0.012	0.80 ± 0.016	0.002
TIA	**0.89 ± 0.008**	**0.85 ± 0.010**	**0.89 ± 0.009**	**0.87 ± 0.007**	**0.86 ± 0.011**	–

The values in bold are observations from the proposed model.

**Table 5:** Results of TIA and baselines on the Amazon MTurk dataset.

Method	Accuracy	Precision	Recall	F1-score	AUC-ROC	*p*-value
MV	0.72 ± 0.019	0.70 ± 0.021	0.73 ± 0.020	0.71 ± 0.018	0.70 ± 0.022	<0.001
DLC	0.77 ± 0.016	0.74 ± 0.018	0.79 ± 0.017	0.76 ± 0.015	0.75 ± 0.019	<0.001
ALKT	0.81 ± 0.013	0.77 ± 0.015	0.82 ± 0.014	0.79 ± 0.012	0.78 ± 0.016	0.001
DS	0.78 ± 0.017	0.76 ± 0.019	0.80 ± 0.018	0.78 ± 0.016	0.76 ± 0.020	<0.001
BWT	0.84 ± 0.010	0.82 ± 0.012	0.85 ± 0.011	0.83 ± 0.009	0.82 ± 0.013	0.005
LCTI	0.80 ± 0.014	0.78 ± 0.016	0.81 ± 0.015	0.79 ± 0.013	0.77 ± 0.017	0.001
CWV	0.79 ± 0.015	0.76 ± 0.017	0.80 ± 0.016	0.78 ± 0.014	0.76 ± 0.018	<0.001
CRMT	0.83 ± 0.011	0.81 ± 0.013	0.84 ± 0.012	0.82 ± 0.010	0.81 ± 0.014	0.007
SWT	0.84 ± 0.012	0.82 ± 0.013	0.85 ± 0.011	0.83 ± 0.010	0.82 ± 0.015	0.005
TIA	**0.88 ± 0.008**	**0.88 ± 0.010**	**0.90 ± 0.009**	**0.87 ± 0.007**	**0.89 ± 0.011**	–

The values in bold are observations from the proposed model.

Similarly, consistent patterns are observed on the WikiSQL and Amazon MTurk datasets, where TIA achieves an accuracy of 89% and 88%, respectively. The detailed comparison of accuracy, precision, recall, F1-Score, and AUC-ROC across the datasets are given in Tables 4 and 5. The high F1-Scores achieved by TIA validate the ability to dynamically evaluate and adjust contributor reliability scores. This ensures that the most accurate and trustworthy contributions are prioritized, thereby enhancing the truth inference outcomes.

Another notable performance is given by the SWT and BWT, both achieving comparable accuracy. This highlights their ability to capture semantic and contextual information effectively. Even without task-specific fine-tuning, both SWT and BWT significantly improve the accuracy and reliability of truth inference compared to traditional methods. Nevertheless, TIA’s integration of task-specific fine-tuning, domain adaptation, and contributor reliability computation yields robust and generalizable performance across all datasets.

In addition, the p-values confirm that TIA consistently achieves statistically significant improvements over all the baseline methods across all datasets. The p-values are below the significance threshold of 0.05 for all comparisons. Also, strong statistical significance (*p* < 0.001) is observed when comparing TIA with traditional methods such as MV and DS. Moreover, for the strongest baseline methods such as CRMT, BWT, and SWT, the improvements remain statistically significant (*p* < 0.05), which confirms the effectiveness of the proposed approach. This statistical validation further strengthens our conclusion that integrating NLP with Swin transformers and transfer learning provides consistent and reliable performance improvements for crowdsourcing truth inference.

**Table 6:** Performance comparison of TIA and various baseline methods under various noisy conditions.

Noise level	Model	Accuracy	Precision	Recall	F1-score
No noise	MV	0.84	0.83	0.85	0.84
	DLC	0.86	0.85	0.87	0.86
	ALKT	0.87	0.86	0.88	0.87
	DS	0.86	0.85	0.87	0.86
	LCTI	0.89	0.88	0.90	0.89
	CWV	0.88	0.87	0.89	0.88
	CRMT	0.89	0.88	0.89	0.89
	TIA	**0.90**	**0.89**	**0.91**	**0.90**
10%	MV	0.81	0.80	0.82	0.81
	DLC	0.83	0.82	0.84	0.83
	ALKT	0.84	0.83	0.85	0.84
	DS	0.83	0.82	0.84	0.83
	LCTI	0.87	0.86	0.88	0.87
	CWV	0.86	0.85	0.87	0.86
	CRMT	0.86	0.85	0.87	0.87
	TIA	**0.88**	**0.87**	**0.89**	**0.88**
20%	MV	0.78	0.77	0.79	0.78
	DLC	0.81	0.80	0.82	0.81
	ALKT	0.82	0.81	0.83	0.82
	DS	0.80	0.79	0.81	0.80
	LCTI	0.85	0.84	0.86	0.85
	CWV	0.84	0.83	0.85	0.84
	CRMT	0.86	0.85	0.87	0.86
	TIA	**0.87**	**0.86**	**0.88**	**0.87**
30%	MV	0.75	0.74	0.76	0.75
	DLC	0.78	0.77	0.79	0.78
	ALKT	0.80	0.79	0.81	0.80
	DS	0.78	0.77	0.79	0.78
	LCTI	0.83	0.82	0.84	0.83
	CWV	0.82	0.81	0.83	0.81
	CRMT	0.83	0.82	0.83	0.82
	TIA	**0.85**	**0.84**	**0.86**	**0.85**
40%	MV	0.72	0.71	0.73	0.72
	DLC	0.76	0.75	0.77	0.76
	ALKT	0.78	0.77	0.79	0.78
	DS	0.76	0.75	0.77	0.76
	LCTI	0.81	0.80	0.82	0.81
	CWV	0.80	0.79	0.81	0.80
	CRMT	0.81	0.80	0.82	0.81
	TIA	**0.83**	**0.82**	**0.84**	**0.84**

The values in bold are observations from the proposed model.

Finally, to further assess the robustness of the proposed method TIA, a comparative analysis under varying levels of noise is performed. In real-world crowdsourcing scenarios, data often contains noise due to the diverse skill levels of contributors and potential errors in their submissions which results in inconsistent labeling, textual perturbations, and missing information. To simulate this, we have introduced controlled noise in the form of random label flips, dropped annotations, and textual input noise, across the dataset. A comprehensive comparison of TIA with the baselines under identical noise conditions is performed.

For fair comparison, ALKT incorporates a noise-aware likelihood function in its probabilistic inference pipeline. Further, in DLC the CNN layers are fine-tuned using noisy data to simulate real-world uncertainty. Also a dropout is introduced during training to increase robustness and improve generalization. BWT and SWT are excluded from this comparison, as they are designed for semantic-level text-based truth inference without explicit noise handling.

The results demonstrate that TIA, particularly when enhanced with regularization techniques, exhibits significant robustness against noisy data. At 30% noise, TIA maintains an F1-Score of 0.85, while CWV, and CRMT though competitive, drop to 0.81 and 0.82, respectively. At 40% noise levels, TIA achieves an F1-Score of 0.84, compared to 0.81 for the best baseline methods. This confirms that TIA exhibits better noise resistance and performance stability compared to the baseline methods.

The implications of these results demonstrate TIA’s practical viability for real-world crowdsourcing deployment. This noise tolerance addresses the main challenges including high noise levels that simulate coordinated spammer attacks, moderate noise that represents mixed-quality contributor pools, and low noise that reflects natural annotation errors. Furthermore, the regularization techniques, such as L2 regularization and dropout, increase the model’s ability to mitigate the effects of noise, while preventing overfitting to noisy inputs. This demonstrates that our model effectively handles noisy data, which is a critical requirement to ensure reliable truth inference in real-world scenarios. The results are presented in Table 6, which show that TIA provides a reliable solution for crowdsourcing environments where data quality varies significantly and malicious contributors may be present.

**Fig. 4:**
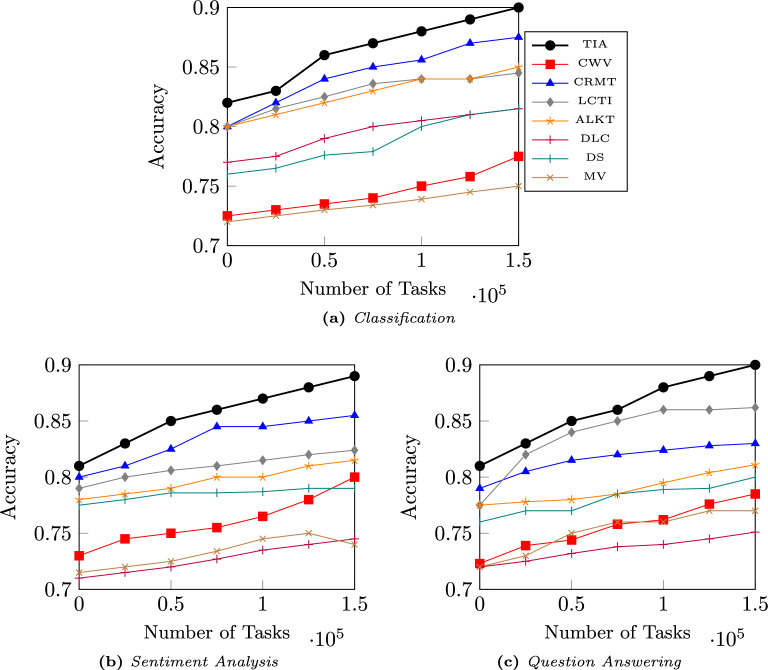
Results of comparison between TIA and baseline methods across different dataset sizes for various type of tasks, in terms of accuracy.

### Scalability

Figure 4 presents the results of the proposed method TIA compared to the baseline methods on varying dataset sizes. The comparison is performed on three different task types, namely, classification, sentiment analysis, and question answering (QA). The x-axis denotes the dataset size in terms of the number of tasks, and the y-axis represents the accuracy of the model. The comparison is performed for three types of tasks, namely classification, sentiment analysis, and question answering (QA). It is observed that TIA consistently outperforms the baseline methods across all three types of tasks, especially as the dataset size grows, which demonstrates the scalability of the proposed approach.

For classification tasks, TIA achieves accuracy ranging from 0.82 to 0.90, and for sentiment analysis tasks, it shows an improvement from 0.81 to 0.89 as the dataset size increases. Similarly, for QA tasks, TIA shows an increase in accuracy from 0.81 to 0.90. These results confirm that the proposed Swin transformer-based model not only performs well with larger datasets but also maintains high accuracy across various types of crowdsourcing tasks. This highlights both the scalability of TIA in handling large-scale crowdsourcing datasets and its generalizability across different task domains.

**Table 7:** Percentage improvements of TIA compared to the baselines across the three datasets.

Dataset	Accuracy (%)	F1-Score (%)	AUC-ROC (%)
Figure Eight	+ 2.4	+ 3.5	+ 3.6
WikiSQL	+ 8.5	+ 7.4	+ 8.9
MTurk	+ 4.8	+ 4.8	+ 8.5
Average	**+ 5.2**	**+ 5.23**	**+ 7.0**

The values in bold are observations from the proposed model.

Table 7 shows percentage improvements of TIA over the baseline methods for each dataset across all evaluation metrics. It is noted that TIA demonstrates consistent improvements ranging from 2.4% to 8.5%, with an average improvement of 5.2% in accuracy, 5.23% in F1-score, and 6.6% in AUC-ROC. The results validate the robust performance improvement of TIA across diverse crowdsourcing tasks and datasets.

### Ablation study

We analyze the impact of different components on the performance of our truth inference model through an ablation study focused on key components. The aim of this study is to understand the individual contributions of these components to the overall performance of the model. The key components evaluated in this study are (1) the use of different NLP techniques, (2) the choice of transfer learning approaches, (3) the inclusion of specific dataset features, (4) the application of regularization techniques, and (5) the model’s robustness under noisy conditions. For each component, we compare the performance of the baseline model with variants where the specific component is either removed or modified.

We compare the performance of our model using two different NLP techniques, namely TF-IDF and word embeddings. From the results, we observe that the TF-IDF approach performs slightly better than word embeddings. This improvement may be attributed to TF-IDF’s ability to emphasize term frequency, which is particularly beneficial for tasks where specific terms are more indicative of the correct label. However, the word embeddings provide a more nuanced representation of the text, capturing semantic relationships between words, which is also valuable for certain tasks.

Next, in the transfer learning approaches, we compare the performance of a pre-trained Swin transformer model with a custom transformer (CT) model trained from scratch. The pre-trained Swin transformer benefits from the linguistic and contextual knowledge gained from large-scale pre-training, allowing it to generalize better to specific crowdsourcing tasks compared to the CT model. The pre-trained Swin transformer outperforms the CT model, showing an average improvement of 3% in F1-Score. This highlights the importance of leveraging pre-trained models for specific task adaptations.

Additionally, we study the impact of additional features such as metadata (e.g., timestamp, user information) and linguistic features (e.g., sentence length, syntactic complexity). It is observed that the inclusion of metadata leads to a marginal improvement in performance, while linguistic features do not significantly impact the results. This suggests that while certain contextual information improves the model’s ability to infer truths, its effectiveness may be constrained by the nature of the tasks and the quality of the available metadata.

**Table 8:** Ablation study of the proposed model.

Component	Precision	Recall	F1-score
NLP techniques			
TF-IDF	**0.85**	**0.78**	**0.81**
Word embeddings	0.82	0.76	0.79
Transfer learning approach			
Pre-trained Swin transformer	**0.88**	**0.82**	**0.85**
CT Model	0.85	0.79	0.82
Dataset features			
Baseline with metadata	0.79	0.75	0.78
Baseline with Linguistic Features	0.78	0.76	0.77
Baseline (with both metadata and linguistic features)	**0.79**	** 0.77**	**0.78**

The values in bold are observations from the proposed model.

Moreover, we incorporate regularization techniques, such as L2 regularization and dropout, to assess their impact on performance. The results indicate that regularization plays a crucial role in preventing overfitting, particularly in complex and large-scale datasets. Regularization is used to prevent overfitting by penalizing large weights, thereby encouraging the model to learn more generalized patterns from the data. In our experiments, we have observed that the inclusion of regularization led to a notable improvement in the model’s performance across several metrics. Specifically, regularization contributed to a 2.0% increase in accuracy and a 2.0% improvement in F1-score on average across the dataset. These improvements indicate that the model became better at handling noise and reducing the impact of outlier contributions, which is critical in diverse crowdsourcing environments. Moreover, regularization significantly enhanced the model’s stability during training, reducing variance in performance metrics across different runs. This demonstrates that regularization not only aids in improving accuracy but also in ensuring that the model remains consistent and reliable, even when exposed to varying data quality and complexity. These findings underscore the importance of regularization in refining the model’s capacity to generalize from limited or noisy data, making it an indispensable component in the design of robust truth inference systems. The results of the ablation study are summarized in Tables 8 and 9.

**Table 9:** Performance comparison with and without regularization for the proposed model with method as Swin transformer.

Regularization	Accuracy	Precision	Recall	F1-score
None	0.87	0.86	0.88	0.87
L2 regularization	0.89	0.88	0.90	0.89
Dropout	0.88	0.87	0.89	0.88
L2 regularization + dropout	**0.90**	**0.89**	**0.91**	**0.90**

The values in bold are observations from the proposed model.

### Computational complexity and inference time

Table 10 demonstrates the inference time results of various methods. It is observed that TIA achieves an optimal balance between computational efficiency and accuracy performance. We have evaluated for four key metrics including single task inference time, which measures individual task processing, batch-16 and batch-64 performance showing parallel processing efficiency at different batch sizes, and overall throughput in tasks per second, which represents the maximum processing capacity when running continuous batch processing.

While TIA shows a 10% increase in single task inference time compared to BWT, this modest overhead is justified by substantial accuracy improvements of 6-7% across all datasets. Further, the batch processing efficiency reveals TIA’s scalability advantages, which achieves 86.2ms per task with batch size 64 and throughput of 12 tasks per second, making it suitable for real-time crowdsourcing applications. At 138.5ms per task, TIA can process approximately 25,000 tasks per hour, meeting the throughput requirements of most crowdsourcing platforms while maintaining better accuracy compared to faster but less accurate alternatives. Further, the results validate that TIA’s 8% parameter increase and 10% inference time increase deliver disproportionate accuracy benefits, which demonstrates a favorable performance-to-computation ratio.

**Table 10:** Inference time comparison across various methods.

Method	Single task (ms)	Batch-16 (ms)	Batch-64 (ms)	Throughput (tasks/s)
MV	2.5	1.8	1.5	667
DS	45.2	38.1	35.7	28
DLC	89.4	67.3	59.2	17
ALKT	112.7	84.5	76.1	13
BWT	125.3	89.2	78.4	13
LCTI	98.6	72.1	64.8	15
CWV	87.3	65.7	58.9	17
CRMT	156.7	112.3	98.6	10
SWT	132.1	94.7	82.3	12
TIA	**138.5**	**98.1**	**86.2**	**12**

The values in bold are observations from the proposed model.

### Discussion

The research insights and ablation study emphasize the importance of various components in the proposed truth inference model. The results suggest that the combination of NLP techniques, transfer learning using Swin transformers, and the Swish activation function improves the performance of our model, which highlights their essential role in an effective truth inference model.

One of the core strengths of TIA is the integration of NLP techniques. This integration provides a more nuanced and semantically meaningful interpretation of crowdsourced labels, which improves the accuracy and robustness of the truth inference process. This is particularly beneficial in handling diverse and complex crowdsourcing tasks including unstructured data such as sentiment analysis, and QA. Moreover, through pre-trained embeddings and contextualized representations, TIA captures the subtle linguistic nuances that are not addressed by traditional truth inference approaches.

As described in section “The proposed framework using NLP and transferlearning with Wwin transformer”, the capabilities of Swin transformer to capture the hierarchical and contextual information significantly improves TIA. When combined with the dynamic aggregation algorithm to compute the contributor reliability, this approach helps to maintain consistency while inferring the true labels. The architectural advantages of the Swin transformer proves to be effective for understanding the complex nature of textual data in crowdsourcing tasks. The results confirm that TIA consistently outperforms the baseline approaches with increasing dataset sizes and various task types. Moreover, for structured tasks like classification, the dynamic truth inference algorithm consolidates the contributor inputs, and the Swin transformer with Swish activation provides a deep semantic understanding for unstructured tasks.

Furthermore, the ablation study validates the contribution of each component in the proposed framework. It is observed that excluding the Swin transformer results in a noticeable drop in accuracy, which shows the importance of hierarchical context modeling in improving the quality of the inferred labels. Similarly, it is noted that removing the contributor reliability mechanism reduces the performance in noisy settings, which signifies its role in mitigating inconsistent labeling. Additionally, incorporating regularization techniques, such as L2 regularization and dropout prevents overfitting and ensures that the model generalizes across various datasets and task domains. This generalization capability is essential in real-world crowdsourced data, which often contains high variability and noise. The better performance of TIA is attributed to the combination of NLP, Swin transformer-based transfer learning, Swish activation function, and regularization strategies. This improves the accuracy and resilience of the proposed model to noisy inputs while maintaining adaptability across various types of tasks and crowdsourcing environments.

The Swin Transformer architecture in TIA shows notable performance, hence it is important to discuss its computational characteristics. The hierarchical structure with shifted window attention mechanism and patch merging operations in TIA gives moderate computational overhead compared to the standard transformer architectures. Quantitatively, it has approximately 115M parameters, which represents an 8% increase over the baseline BWT. Similarly, the inference latency increases by approximately 10% compared to BWT. Despite this modest increase in the computational requirements, the performance improvements are substantial, with 2.4% higher accuracy on the Figure Eight dataset, 8.5% on WikiSQL, and 4.85% on MTurk compared to the baselines. This favorable performance-to-computation ratio validates our architectural design choices. Moreover, the hierarchical nature of the Swin Transformer, enabled by the patch merging operations, is crucial for capturing both local context (through window attention) and global dependencies (through shifted windows) in crowdsourced data for accurately inferring truth from contributions with varying reliability. For applications where inference speed is critical, optimization techniques such as model pruning or knowledge distillation can be applied to reduce computational demands while preserving most of the performance benefits.

In summary, the proposed model TIA is a balanced and scalable framework for truth inference, by integrating NLP, Swin transformer, and dynamic aggregation algorithm for deep contextual learning. Its ability to handle both structured and unstructured tasks, along with resilience to noise, makes it a dependable solution for real-world crowdsourcing applications.

## Conclusion

Truth inference strongly impacts crowdsourcing while aggregating the responses of diverse contributors. The problem of truth inference in crowdsourcing systems, and the challenges in improving its accuracy, scalability, and robustness have been studied. We have proposed a truth inference model that integrates NLP techniques and Transfer Learning using the Swin transformer to infer reliable responses from crowdsourced data. The use of hierarchical contextual embeddings and semantic understanding, which is combined with transfer learning enables improved processing of textual data from both structured and unstructured tasks while generalizing across various task domains with minimal training. Comprehensive experiments across multiple datasets demonstrate that the proposed Swin transformer-based model achieves consistent improvements with statistical significance of $p < 0.05$, showing average improvements of $5.2\%$ in accuracy, $5.23\%$ in F1-score, and $7.0\%$ in AUC-ROC. Moreover, the model effectively adapts to various tasks and contributor inputs, and shows improved noise tolerance, maintaining 85% F1-score at 30% noise levels compared to $82\%$ for the best baseline. Even though we have demonstrated the effectiveness of the proposed model across structured tasks and unstructured tasks, it does not address multimodal crowdsourcing tasks that combine different data types, such as image-text pairs or audio-text combinations. Such multimodal tasks are increasingly common in modern crowdsourcing platforms, where workers provide textual descriptions or labels for visual or auditory content. Extending the proposed framework to support multimodal inputs would significantly enhance its practical utility. This could be achieved by integrating vision-language or audio-language models with our Swin Transformer-based approach, which allows the model to jointly reason about multiple modalities while assessing contributor reliability. Additionally, integrating meta-learning techniques could improve the adaptability in dynamically changing crowdsourcing environments.

## Data Availability

The data supporting the findings of this study are available from the corresponding author on request.
